# Development and validation of a novel prognostic model for predicting AMD progression using longitudinal fundus images

**DOI:** 10.1136/bmjophth-2020-000569

**Published:** 2020-10-15

**Authors:** Joshua Bridge, Simon Harding, Yalin Zheng

**Affiliations:** Department of Eye and Vision Science, University of Liverpool, Liverpool, UK

**Keywords:** retina, imaging

## Abstract

**Objective:**

To develop a prognostic tool to predict the progression of age-related eye disease progression using longitudinal colour fundus imaging.

**Methods and analysis:**

Previous prognostic models using deep learning with imaging data require annotation during training or only use a single time point. We propose a novel deep learning method to predict the progression of diseases using longitudinal imaging data with uneven time intervals, which requires no prior feature extraction. Given previous images from a patient, our method aims to predict whether the patient will progress onto the next stage of the disease. The proposed method uses InceptionV3 to produce feature vectors for each image. In order to account for uneven intervals, a novel interval scaling is proposed. Finally, a recurrent neural network is used to prognosticate the disease. We demonstrate our method on a longitudinal dataset of colour fundus images from 4903 eyes with age-related macular degeneration (AMD), taken from the Age-Related Eye Disease Study, to predict progression to late AMD.

**Results:**

Our method attains a testing sensitivity of 0.878, a specificity of 0.887 and an area under the receiver operating characteristic of 0.950. We compare our method to previous methods, displaying superior performance in our model. Class activation maps display how the network reaches the final decision.

**Conclusion:**

The proposed method can be used to predict progression to advanced AMD at some future visit. Using multiple images at different time points improves predictive performance.

**Key messages**

**What is already known about this subject?**

- Previous studies showed that deep learning has great predictive capability for AMD progression when using a single colour fundus image.

**What are the new findings?**

- Using multiple time points allows us to model the temporal aspect of the disease and improves predictive performance.

**How might these results change the focus of research or clinical practice?**

- Future studies should greater consider the rate of disease progression in different patients.

## Introduction

Prognostic models are an essential component of personalised medicine, allowing health experts to predict the future course of disease in individual patients.^1^ Advances in computing power and an abundance of data have allowed for increasingly sophisticated models to be developed. Most developed prognostic models use statistical methods such as logistic regression; these models require prior feature extraction, either manual or automatic,^2^ and are limited in the number of included variables. Feature extraction can be costly and time consuming, especially in imaging data. Deep learning offers the ability to avoid explicit feature extraction, allowing us to develop models without the need for handcrafted features. For this reason, deep learning is especially useful in imaging data. Prognostic deep learning models have been developed in several fields, primarily ophthalmology,^3^ cardiology^4^ and neurology,^5^ and several modalities, including MRI, optical coherence tomography (OCT), colour fundus photography and X-ray.

Current prognostic models that use deep learning to analyse imaging data, either use automatic feature extraction algorithms to extract known features or only consider a single time point. Models developed using feature extraction, train algorithms on annotated images to extract relevant features such as volumes in OCT data; those features are then fed into a traditional statistical model, see Refs. 3 6 7 for examples. Manual feature extraction is time consuming and requires expert readers. Yim *et al*^8^ proposed a method which automatically segments OCT layers before classification. This method outperformed human experts; however, automatic feature extraction requires annotations during training, which is not always available in situations when the features are unknown or difficult to quantify, such as is the case when using colour fundus imaging.

An alternative to explicit feature extraction is to use deep learning to extract features implicitly, such as used by Arcadu *et al*^9^ and Babenko *et al*.^10^ Many models take the previous available image and fit a pretrained convolutional neural network (CNN), with Inception V3^11^ being a popular choice due to its generalisability and high performance in a variety of tasks. This method, unlike the feature extraction method, may be applied to any image even when features are not explicitly known; however, this creates a separate issue, by using only one image, these models may fail to capture the temporal pattern across time points. Most recently, Yan *et al*^12^ used Inception V3 to classify single images combined with genetic factors to predict progression to AMD. They found that images alone provided reasonable performance, and the inclusion of multiple genetic factors increased predictive performance; however, this work still only considered a single time point.

Here, we develop a prognostic model to predict the progression of disease, from longitudinal images. The proposed method is demonstrated on a dataset consisting of 4903 eyes with age-related macular degeneration (AMD), taken from the Age-Related Eye Disease Study (AREDS) dataset.^13^ The method is generalisable to any longitudinal imaging data. We show that by considering the time interval between images and adopting a method from time series analysis, we can provide significantly improved prediction performance.

Our contributions are as follows:

- Propose a novel method to predict the future prognosis of a patient from longitudinal images.
- Introduce interval scaling which allows for uneven time intervals between visits.
- Demonstrate on the largest longitudinal dataset and attain state-of-the-art performance outperforming other state-of-the-art methods.

## Materials and methods

Given images {*X*_*0*_*…, X*_*i*_*…, X*_*N*_} at times {*t*_*0*_*…, t*_*1*_*… t*_*N*_} we wish to predict the diagnosis $y_{N+1}$ at time $t_{N+1}$, where $t_{i+1}-t_{i}=t_{i}-t_{i-1}$ does not necessarily hold, which is common in a clinical setting.

**Figure 1:**
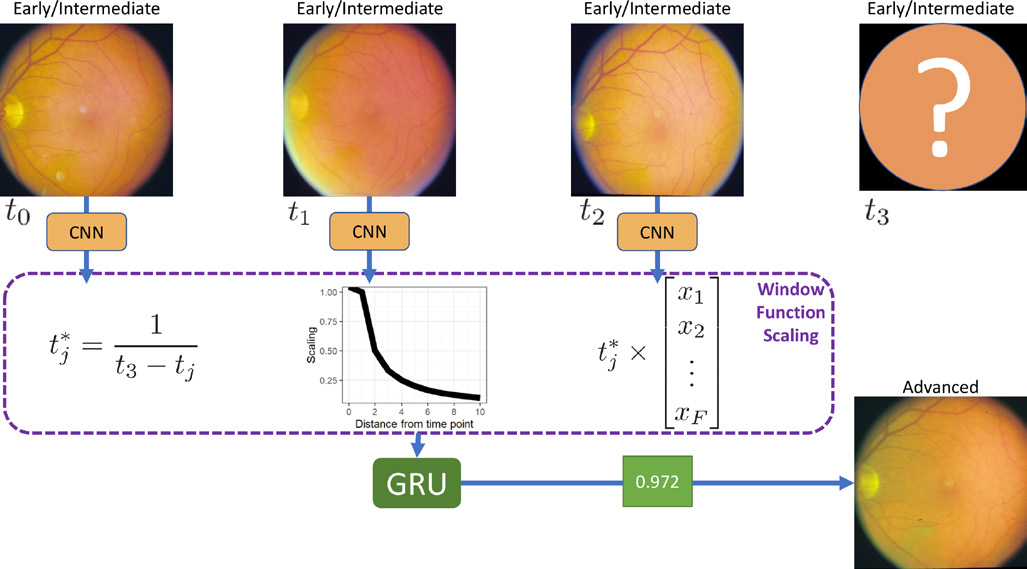
Overview of the proposed method. For each of the T time points, we fit a convolutional neural network (CNN) with shared weights, resulting in a vector of length F, per image. Each vector is multiplied by a corresponding interval scaling. The scaled vectors are combined into a single T×F matrix, and a gated recurrent unit (GRU) with sigmoid activation gives a probability of progression. For simplicity, three time points are shown; this method is extendable to any number of time points.

The proposed method consists of three stages, first, we use a pretrained CNN, namely Inception V3, with shared weights, to reduce each image to a single feature vector. Then, the feature vectors are combined, and an interval scaling is applied to account for the uneven time intervals, this weights the most recent time points as being more important in making the final prediction. Finally, a recurrent neural network classifies the images as progressing or non-progressing. An overview of the proposed framework is shown in figure 1.

### Inception V3

We begin by fine-tuning a pretrained CNN on each image, with shared weights, to extract feature vectors. CNNs are commonly used on imaging data and use multiple convolutional layers to produce feature maps which represent the original image. These feature maps are smaller than the original image which allows a fully connected layer to classify the features, with reasonable computational requirements. In our work, we chose Incpetion V3^11^ pretrained on ImageNet.^14^ InceptionV3 increases accuracy over previous networks while remaining computationally efficient, through the use of factorised kernels, batch normalisation and regularisation. InceptionV3 is considered highly generalisable with a greater than 78.1% accuracy on the ImageNet dataset. The Inception V3 network results in a feature vector of length F=2048 for each image at each time point. This network has previously been used to provide state of the art results in single time point methods,^9 10 12^ and is used here as a feature extractor.

### Interval scaling

To account for uneven time intervals, we implement a triangular window function to create a smoothing model. Whereas in a simple moving average model, the time points are weighted equally, smoothing models weight values closer to $t_{N+1}$ as being more useful in the prediction. For each sequence of images at times, $t_{0},\ldots ,t_{i},\ldots ,t_{N+1},$ where $t_{N+1}$ is the time point that we want to predict at, we rescale each time such that $t_{i}^{*}=1/(t_{N+1}-t_{i})$. The feature vectors for each image are then multiplied by their corresponding time interval scale. This scaling weights the images such that images closer to the time point of interest are considered more important than those observed at further time points, thus allowing the network to account for uneven time intervals.

### GRU prediction

To predict whether the patient will progress to advanced AMD or not, we combine the interval corrected vectors into a T×F matrix, where T corresponds to the number of time points and F is the number of features. We apply a gated recurrent unit (GRU)^15^ with a filter size of 1, resulting in a single value. GRU was chosen as opposed to long short-term memory (LSTM)^16^ units, as GRU is more computationally efficient. LSTM units perform better on longer sequences; however, in this case, we only have three time points.^17^ The sigmoid activation function then scales the value between 0 and 1.

### Data

Data consist of colour fundus images taken from the AREDS,^13^ the most extensive clinical study into AMD. All data used are available from dbGap (accession: phs000001.v3.p1). This was a retrospective study. Patients or the public were not involved in the design, or conduct, or reporting, or dissemination plans of our research. Patient consent is detailed in AREDS report no. 1.^13^

AMD is a leading cause of vision loss worldwide.^18^ There are two main stages of AMD, early/intermediate, defined by small-sized to medium-sized drusen, and advanced, defined by geographic atrophy (GA) or neovascularisation (nAMD).^13^ Drusen can be observed as yellow-white lipid deposits under the retina, varying greatly in size and morphology.^19^ The exact causes of AMD are unknown; however, studies have shown that smoking and genetics are significant risk factors.^20^ Risk factors for progression from early/intermediate to advanced AMD are also unknown; however, there is evidence that drusen and optic disk characteristics are important.^21 22^ Vision loss can be avoided with interventions such as anti–vascular endothelial growth factor (anti-VEGF) treatment; however, disease progression and the need for treatment are often hard to predict.^23^ This highlights the need for accurate prognostic models.

We extracted 4903 eyes, from 2702 patients, which had a minimum of four visits, complete with images and diagnoses at each visit, with no diagnosis of advanced AMD during the first three visits. The most recent visits meeting these criteria were always used. Advanced AMD was defined as either Central GA, nAMD, or both GA and nAMD. We used the last visit as ground truth to make our prediction based on the first three visits. Of the 4903 included eyes, 453 (9.2%) progressed to advanced AMD.

**Figure 2:**
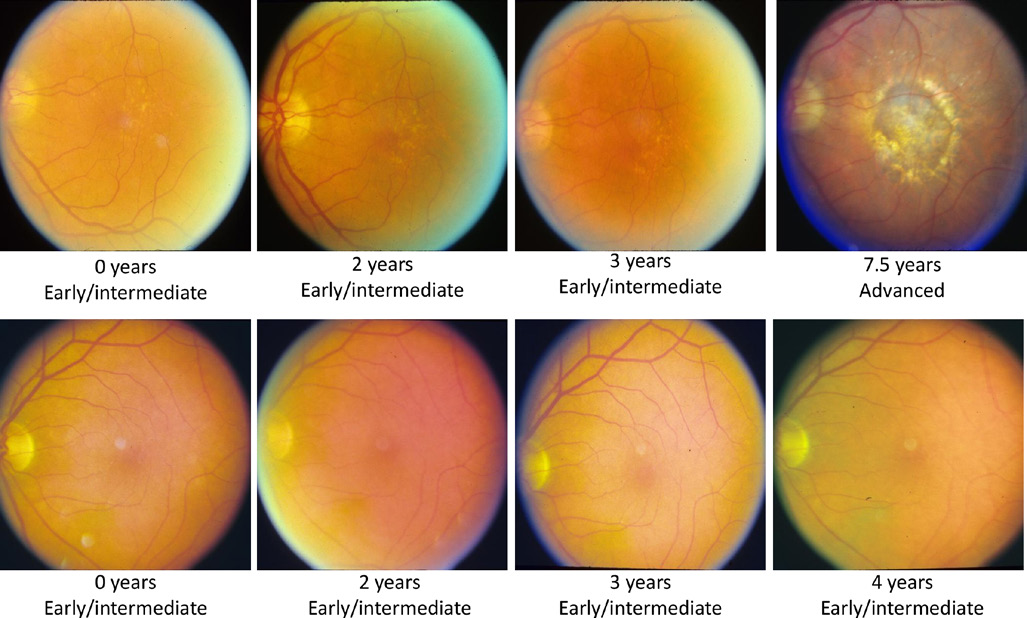
Sample images from a progressing patient (top) and non-progressing patient (bottom). The first three images show early/intermediate age-related macular degeneration (AMD), while the fourth image shows whether they progressed to advanced AMD or not.

We randomly split the data into 60% training (2942 eyes, 272 progressing), 20% validation (981 eyes, 91 progressing), and 20% testing (980 eyes, 90 progressing) datasets. To reduce the possibility of data leakage, patients with both eyes included were kept within the same data split. Example images are given in figure 2.

### Preprocessing

Any images in the dataset where the patient had already progressed to advanced AMD, or without the required three previous images plus a fourth prediction image for prediction, were excluded. The images were automatically cropped by first calculating the difference between the original image and the background colour, an offset was added to the difference, and the bounding box was calculated from this. Image values were rescaled from between 0 and 255 to between 0 and 1. All images were resized to 256×256 pixels to reduce computational requirements. Right eye images were flipped, such that the optic disc on all images was located on the left. No prior feature extraction or segmentation/registration is required, such that our method is as generalisable to other diseases and modalities as possible. All preprocessing was automated, with no subjective human input required.

### Computing

All analyses were carried out on a Linux machine with a Titan X 12 GB GPU and 32 GB of memory. Deep learning was conducted in Python V.3.7 using the Keras V.2.2.4 library^24^ with TensorFlow^25^ as the base library. Code is available on request. CIs were calculated using R V.3.4.4,^26^ with the pROC package.^27^

Optimisation was carried out with the Adam optimizer^28^ with an initial learning rate of 0.0001. We used binary cross-entropy as the loss function. If the loss did not improve after 10 epochs, then the learning rate was reduced to two-thirds. Model checkpoints and early stopping prevented overfitting, with the best model being picked according to the validation loss.

### Metrics

We evaluate model performance using the commonly used area under the receiver operating characteristic curve (AUC),^29^ optimal sensitivity and optimal specificity, determined by Youden’s index. To assess whether the difference in these measures between models is significant, we construct CIs. De Long’s method^30^ is used to construct CIs for AUC, and bootstrapping with 2000 samples is used for sensitivity and specificity to calculate 95% CIs. Results from De Long’s test^30^ are also reported.

## Results

In order to evaluate the performance, the proposed method is demonstrated on a dataset of AMD images with two and three time points and compared with a single time point method. For comparisons, the most recent visits were used.

Results are reported using two and three time points with our method, to assess the benefit of adding additional time points. We compare our results with the image only method used by Yan *et al*^12^ using a single time point. Taking the last available image, we fine-tune InceptionV3^11^ pretrained on ImageNet^14^ to classify as progression or no progression.

The proposed method using three time points achieves an AUC, optimal sensitivity and optimal specificity of 0.950 (0.923 to 0.977), 0.878 (0.810 to 0.945), and 0.887 (0.866 to 0.907), respectively; this is a significant improvement over the single time point method which had AUC, sensitivity and specificity of 0.857 (0.823 to 0.890), 0.867 (0.796 to 0.937) and 0.760 (0.731 to 0.788). These results show a statistically significant increase in AUC and specificity and a non-significant increase in sensitivity when using the proposed three time point method over the previous single time point methods. De Long’s test gave a p value<0.0001, indicating a significant difference in AUCs. This significant increase in specificity without a loss in sensitivity shows our model can reduce false positives without increasing false negatives, over the previous model.

**Figure 3:**
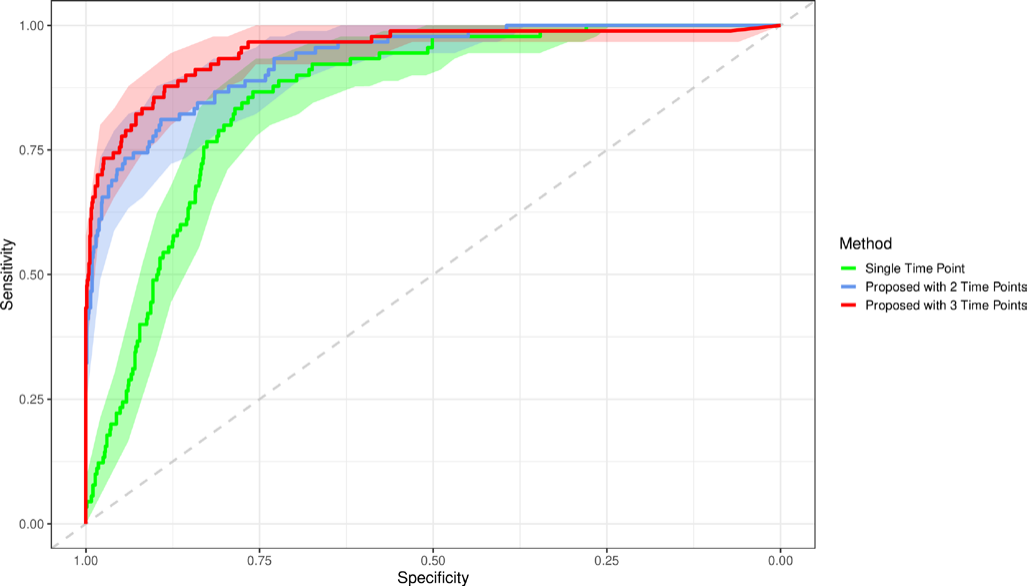
Receiver operating characteristic curve for the single time point InceptionV3 method, the proposed method with two time points and the proposed time point with three time points. Increasing the number of time points appears to increase the area under the curve. Faded bands show 95% CIs.

The method using two time points gave an AUC, sensitivity and specificity of 0.932 (0.905 to 0.958), 0.811 (0.730 to 0.892) and 0.892 (0.872 to 0.913). The three time point method had a non-significant increase over two time points. This may indicate that in this example, using more than two time points does not add any significant predictive value. Results are presented in table 1, and the receiver operating characteristic is shown in figure 3. Experiments without interval scaling were also conducted and showed a significant decrease in performance.

**Table 1:** Area under the receiver operating characteristic (AUC) with 95% CIs constructed by De Long’s method

	AUC	Sensitivity	Specificity
Yan *et al*^12^	0.857 (0.823 to 0.890)	0.867 (0.796 to 0.937)	0.760 (0.731 to 0.788)
Proposed method (two time points)	0.932 (0.905 to 0.958)	0.811 (0.730 to 0.892)	0.892 (0.872 to 0.913)
Proposed method (three time points)	0.950 (0.923 to 0.977)	0.878 (0.810 to 0.945)	0.887 (0.866 to 0.907)

Area Under the Receiver Operating Charteristic Curve (AUC) with 95% confidence intervals (CIs) constructed using De Long's method. Sensitivity and specificity with 95% CIs constructed by bootstrapping with 2000 samples.

### Class activation maps

**Figure 4:**
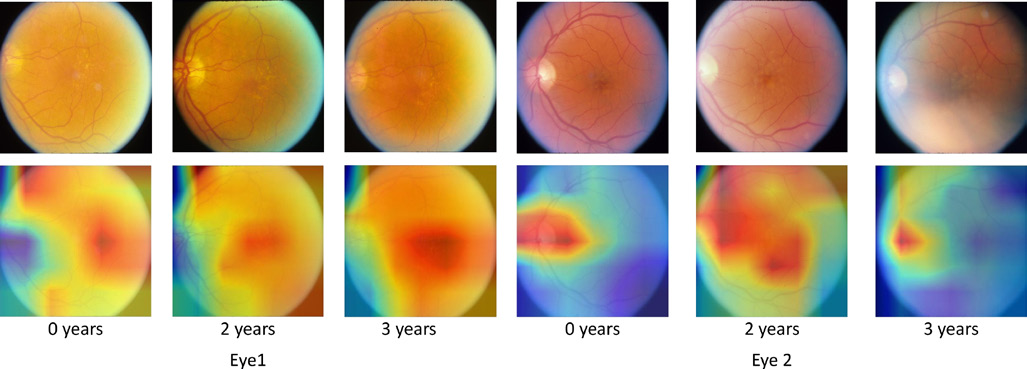
Class activation maps show the areas that the network finds useful in making the prediction. Original images are also shown for reference. The network correctly identifies areas of interest in age-related macular degeneration progression. In blurred images, drusen are difficult to see; the class activations show that the network uses the optic disk in to reach decisions in this case. All example images are taken from the testing dataset.

To determine if our network is identifying the correct features and to reduce the blackbox nature of deep learning, we create class activation maps^31^ for each time point. We altered the top of the network slightly to achieve this, adding a dense layer after the GRU layer. While this altered network showed no significant change in predictive performance, it increased the network size by around a factor of 2. The class activation maps are shown in figure 4, alongside original images for comparison.

The class activation maps show that areas with high concentrations of drusen are considered relevant by the network; this is expected and shows that our network is identifying the correct features. In some images, the optic disk is also highlighted, confirming that optic disk characteristics are indeed important factors in AMD progression, as observed previously.^21 22^ In images where drusen are challenging to see, the network appears to use the optic disk solely in making a prediction. It is also interesting to note that the network seems to be surer of the area of interest in images that are closer to the prediction time point. In a clinical setting, these maps may are useful when justifying the prediction.

## Discussion

In this work, we proposed a novel deep learning prognostic model to predict the future onset of disease. The proposed method addresses the challenge of analysing multiple longitudinal images with uneven time points, without the need for prior image annotation. Introducing an interval scaling was shown to improve performance over a single time point method significantly. We show that by taking into account the varying times between observed images, we can significantly improve the performance of a longitudinal prognostic model. Our method provides a statistically significant increase in specificity, which is critical in contexts such as screening. Our method uses time intervals meaning we can extend the interval to the observed outcome to predict further into the future; this is useful in a screening context. Future work is required to assess the generalisability of the proposed method to other diseases and to extend its use to a screening context. The disciform changes highlighted by the class activations maps may be similar to glaucoma; patients with glaucoma could potentially be wrongly classified. Glaucoma diagnosis was not available in this study, but could be used as a covariate in future work to prevent a wrong prognosis and to improve model performance. The proposed method is applicable to many other retinal diseases such as glaucoma and diabetic retinopathy, and could even be used outside of ophthalmology. Our work aims to predict progression at a future visit, whenever that is; future work is needed to assess the models use for dynamic prediction.

## References

[1] Steyerberg EW, Moons KGM, van der Windt DA, et al Prognosis research strategy (progress) 3: prognostic model research. PLoS Med 2013;10:e1001381–e81. 10.1371/journal.pmed.1001381 23393430 PMC3564751

[2] Bohn E, Tangri N, Gali B, et al Predicting risk of mortality in dialysis patients: a retrospective cohort study evaluating the prognostic value of a simple chest X-ray. BMC Nephrol 2013;14:263. 10.1186/1471-2369-14-263 24289833 PMC4219436

[3] de Sisternes L, Simon N, Tibshirani R, et al Quantitative SD-OCT imaging biomarkers as indicators of age-related macular degeneration progression. Invest Ophthalmol Vis Sci 2014;55:7093–103. 10.1167/iovs.14-14918 25301882

[4] Kwon J-M, Kim K-H, Jeon K-H, et al Artificial intelligence algorithm for predicting mortality of patients with acute heart failure. PLoS One 2019;14:e0219302. 10.1371/journal.pone.0219302 31283783 PMC6613702

[5] Hilario A, Sepulveda JM, Perez-Nuñez A, et al A prognostic model based on preoperative MRI predicts overall survival in patients with diffuse gliomas. AJNR Am J Neuroradiol 2014;35:1096–102. 10.3174/ajnr.A3837 24457819 PMC7965146

[6] Leng T, de Sisternes L, Chen Q Automated prediction of AMD progression from quantified SD-OCT images. Investigative Ophthalmology & Visual Science 2013;54:4150–50.

[7] Niu S, de Sisternes L, Chen Q, et al Fully automated prediction of geographic atrophy growth using quantitative spectral-domain optical coherence tomography biomarkers. Ophthalmology 2016;123:1737–50. 10.1016/j.ophtha.2016.04.042 27262765

[8] Yim J, Chopra R, Spitz T, et al Predicting conversion to wet age-related macular degeneration using deep learning. Nat Med 2020;26:892–9. 10.1038/s41591-020-0867-7 32424211

[9] Arcadu F, Benmansour F, Maunz A, et al Deep learning algorithm predicts diabetic retinopathy progression in individual patients. NPJ Digit Med 2019;2:92. 10.1038/s41746-019-0172-3 31552296 PMC6754451

[10] Babenko B, Balasubramanian S, Blumer KE, et al Predicting progression of age-related macular degeneration from fundus images using deep learning. arXiv preprint arXiv 2019;190405478.

[11] Szegedy C, Vanhoucke V, Ioffe S, et al Rethinking the inception architecture for computer vision.2818-26.

[12] Yan Q, Weeks DE, Xin H, et al Deep-learning-based prediction of late age-related macular degeneration progression. Nat Mach Intell 2020;2:141–50. 10.1038/s42256-020-0154-9 32285025 PMC7153739

[13] Age-Related Eye Disease Study Research Group The age-related eye disease study (AREDS): design implications. AREDS report No. 1. Control Clin Trials 1999;20:573–600. 10.1016/s0197-2456(99)00031-8 10588299 PMC1473211

[14] Russakovsky O, Deng J, Su H, et al ImageNet large scale visual recognition challenge. Int J Comput Vis 2015;115:211–52. 10.1007/s11263-015-0816-y

[15] Cho K, Van Merriënboer B, Gulcehre C, et al Learning phrase representations using RNN encoder-decoder for statistical machine translation. arXiv preprint arXiv 2014;14061078.

[16] Hochreiter S, Schmidhuber J Long short-term memory. Neural Comput 1997;9:1735–80. 10.1162/neco.1997.9.8.1735 9377276

[17] Chung J, Gulcehre C, Cho K, et al Empirical evaluation of gated recurrent neural networks on sequence modeling. arXiv preprint arXiv 2014;14123555.

[18] Wong WL, Su X, Li X, et al Global prevalence of age-related macular degeneration and disease burden projection for 2020 and 2040: a systematic review and meta-analysis. Lancet Glob Health 2014;2:e106–16. 10.1016/S2214-109X(13)70145-1 25104651

[19] Williams BM, Burgess PI, Zheng Y Chapter 13 - Drusen and macular degeneration, 2019: 245–72.

[20] Chakravarthy U, Wong TY, Fletcher A, et al Clinical risk factors for age-related macular degeneration: a systematic review and meta-analysis. BMC Ophthalmol 2010;10:31. 10.1186/1471-2415-10-31 21144031 PMC3009619

[21] Law SK, Sohn YH, Hoffman D, et al Optic disk appearance in advanced age-related macular degeneration. Am J Ophthalmol 2004;138:38–45. 10.1016/j.ajo.2004.02.021 15234280

[22] Scheufele TA, McHenry JG, Edwards AO Optic neuropathy and Age–Related macular degeneration. Investigative Ophthalmology \& Visual Science 2004;45:1627–27.

[23] Kovach JL, Schwartz SG, Flynn HW, et al Anti-Vegf treatment strategies for wet AMD. J Ophthalmol 2012;2012:1–7. 10.1155/2012/786870 PMC3317200 22523653

[24] Chollet F et al Keras, 2015.

[25] Abadi M, Agarwal A, Barham P, et al Tensorflow: large-scale machine learning on heterogeneous distributed systems. arXiv preprint arXiv 2016;160304467.

[26] R Foundation for Statistical Computing R: A language and environment for statistical computing. [program. R Foundation for Statistical Computing, 2019.

[27] Robin X, Turck N, Hainard A, et al pROC: an open-source package for R and S+ to analyze and compare ROC curves. BMC Bioinformatics 2011;12:77. 10.1186/1471-2105-12-77 21414208 PMC3068975

[28] Kingma DP, Ba J Adam: a method for stochastic optimization. arXiv preprint arXiv 2014;14126980.

[29] Harrell FE, Califf RM, Pryor DB, et al Evaluating the yield of medical tests. JAMA 1982;247:2543–6. 7069920

[30] DeLong ER, DeLong DM, Clarke-Pearson DL Comparing the areas under two or more correlated receiver operating characteristic curves: a nonparametric approach. Biometrics 1988;44:837–45. 10.2307/2531595 3203132

[31] Zhou B, Khosla A, Lapedriza A, et al Learning deep features for discriminative localization.2921-29.

